# Optimization of polymerase chain reaction for the identification of Roe deer, Saiga, and Siberian stag living in Kazakhstan

**DOI:** 10.14202/vetworld.2022.2067-2071

**Published:** 2022-08-26

**Authors:** Kanatbek Mukantayev, Darkhan Kanayev, Sholpan Zhumabekova, Alexander Shevtsov, Kanat Tursunov, Kasim Mukanov, Yerlan Ramankulov

**Affiliations:** 1 Laboratory of Immunochemistry and Immunobiotechnology, National Center for Biotechnology, Nur-Sultan, Kazakhstan; 2 Laboratory of Applied Genetics, National Center for Biotechnology, Nur-Sultan, Kazakhstan

**Keywords:** Argali, cytochrome b, identification, Roe deer, Saiga, Siberian stag

## Abstract

**Background and Aim:**

One of the reasons for the decline in the number of wild species of artiodactyls is poaching and the illegal trading of animal products. Molecular genetic identification of animals from a biological sample effectively proves poaching cases and illegal trade of animal products. This study aimed to develop a polymerase chain reaction (PCR) test that allows for species identification of artiodactyl animals that are most often subject to poaching.

**Materials and Methods:**

Genomic DNA was extracted from meat and blood samples of animals killed by poachers using commercial kits. Three pairs of primers were designed and used to amplify the cytochrome b gene fragment of Roe deer, Saiga antelope, and Siberian stag.

**Results:**

The proposed protocol allows amplification of specific PCR products of 542 bp with Roe deer DNA, 587 bp with Saiga DNA, and 525 bp with Siberian stag DNA. Specificity analysis showed no cross activity with DNA from other animal species. The detection limit of PCR ranged from 15.6 pg to 1.9 pg of DNA in 25 mL of the reaction mixture.

**Conclusion:**

Sequencing the amplified products and subsequent comparison with the corresponding reference sequence showed a similarity ranging from 99.99% to 100%. The PCR based on the developed primers demonstrated high sensitivity and specificity when using DNA from homogeneous and heterogeneous animals.

## Introduction

The conservation of endangered species of artiodactyl animals such as Roe deer, Saiga, and the Siberian stag is an urgent issue in the Republic of Kazakhstan. The main reasons for the decline in the number of these species are the expansion of human economic activity, uncontrolled hunting, and trading of wild animal products [1]. One effective approach to reducing the negative impact of poaching and illegal trade in wild fauna products is the use of molecular genetic methods for animal identification based on a biological sample. For instance, the use of molecular genetic methods to study biological samples has ensured compliance with legislation against poaching and illegal trade in manatees [2], wild Caucasian tur (*Capra caucasica*) [3], and greater one-horned rhinoceros [4]. Animal identification based on biological samples was carried out by the traditional method of morphological determination of animal remains. In forensic medical examination, immunological methods were widely used, including the procedure for determining the antigen fraction using specific antibodies. The main disadvantages of these methods were the complexity of morphological identification of animals by tissue pieces, low stability of antibodies under environmental conditions, and lack of specificity at the level of animal species [5, 6].

Molecular genetic methods of animal species identification based on biological samples are the most highly specific and reliable. The development of molecular genetic methods for animal identification made it possible to use biological samples such as blood, hair (fur), feathers, skin, muscle samples, and feces [7–9]. At present, for species identification of animals by a biological sample, the restriction fragment length polymorphism of the polymerase chain reaction (PCR/RFLP), simple sequence repetition (SSR), and single-nucleotide polymorphism (SNP) are used. PCR/RFLP includes three steps: DNA isolation, PCR, and cleavage of PCR products using restriction enzymes [10, 11]. The main disadvantage of these methods is the inability to distinguish mixed samples from possible unknown species. This problem is solved by sequencing specific regions amplified with universal primers. The resulting sequences are compared with reference sequences using the BLAST search algorithm [12, 13]. The SNP has become one of the most reliable markers for identifying animals from a biological sample since these mutations are numerous and widespread in the genomes of most animal species. These markers are not only reliable and sensitive but also relatively inexpensive. Using the SNP fragment of the D-loop gene of mitochondrial DNA effectively identified 10 wild African animal species that were affected by illegal hunting [14]. The cytochrome b (Cyt b) gene is widely used to identify vertebrate species. The multicopy nature of the mitochondrial genome, the high interspecies variability of the Cyt b gene, and the use of a large database of nucleotide sequences in phylogenetic relations, as a result, made it possible to use this marker in the development of PCR tests for species identification of animals [8, 15, 16]. The presence of conservative regions of the genome allows using universal primers for identifying an animal species by the cytochrome gene. Ideal for identifying an animal is primers that amplify regions of a gene that is sufficiently different between animal species. Equally important is the availability of the size of the amplified gene fragment for sequencing reaction [12]. Several primers have been developed to identify animal species based on the first 400 bp of the cytochrome gene [17].

Kazakhstan is implementing several programs to preserve the biological diversity of animal and plant fauna: Reserves are being created and legislation is being tightened for shooting and harming wildlife. Despite this, poaching cases are registered annually, and unfortunately, many of them remain unproven due to the limited evidence base. This is because species identification of animals is still carried out using immunological methods or based on morphological differences, which does not allow us to establish the species identity as a pure one, especially if poachers get rid of hooves, skins, heads, etc. Consequently, this study aimed to develop a PCR test that allows for species identification of artiodactyl animals that are most often subject to poaching.

## Materials and Methods

### Ethical approval

All of the samples were obtained from wild animals killed by poachers. All samples from domestic animals were obtained during the necessary diagnostic procedures by veterinary staff; therefore, no ethics committee approval was necessary.

### Study period, location, animal, and sample collection

The study was conducted from January 2021 to December 2021, with target samples from the northern part of Kazakhstan. For DNA extraction, tissues of Roe deer (*Capreolus capreolus*), Saiga antelope (*Saiga tatarica*), Siberian stag (*Cervus elaphus sibiricus*), argali (*Ovis ammon*), sheep (*Ovis aries*), and cow (*Bos taurus*) were used. The species of artiodactyl animals of Kazakhstan used in this study, as well as the type and number of samples analyzed, are listed in Table-1.

**Table-1:** Classification of wild and domestic artiodactyl animals of Kazakhstan as well as the type and number of samples.

Class	Order	Family	Species	Common name	Specimen type	Number of specimens
Mammals	Artiodactyls	Deer	*Capreolus pygargus*	Roe deer	Meat	6
			*Cervus elaphus sibiricus*	Siberian stag	Blood	1
		Bovid	*Saiga tatarica*	Saiga	Bone	1
			*Ovis ammon*	Argali	Meat	1
			*Ovis aries*	Sheep	Meat	3
			*Bos taurus*	Cattle	Meat	3

Genomic DNA was extracted from meat samples of animals killed by poachers using the GeneJET Genomic DNA Purification Kit (Thermo Scientific, USA). DNA extraction from blood was performed using QIAamp DNA Blood Kits (Qiagen, Germany), according to the manufacturer’s instructions.

### Primer design

The search for sequences homologous to target genes containing consensus motifs was performed using the National Center for Biotechnology Information (NCBI) web resource (https://www.ncbi.nlm.nih.gov/genome). Sequence alignment was carried out using BioEdit Sequence Alignment Editor 1997–2013 software (https://bioedit.software.informer.com/7.2/) [18]. Primer design and estimation of the probability of formation of secondary structures (such as hairpins and dimers) were calculated using the PrimerSelect program (DNASTAR). The specificity of the primers, as well as the melting point, was tested *in silico* using the web service Primer-BLAST (http://www.ncbi.nlm.nih.gov/tools/primer-blast). The designed primers are shown in Table-2.

**Table-2:** Primers for species identification of Saiga, Roe deer, and Siberian stag.

Species	Common name	Accession number	Primers	Amplicon size
*Capreolus pygargus*	Roe deer	KT964433.1	for 5’CATGGTGAAACTTTGGCTCTC 3’ rev 5’TGTTGGGTTGTTTGATCCTGTTTC 3’	542
*Saiga tatarica*	Saiga	JX177502.1	for 5’ GACACAGCAACAGCATTCCACTCT 3’ rev 5’GGGTCTCCAAGCAGGTCT 3’	587
*Cervus elaphus*	Siberian stag	NC_007704.2	for 5’CGGGGCATCAATATTTTTCATCTG 3’ rev 5’ TTTGCTGGGGTGTAGTTATCTGGA 3’	525

### Polymerase chain reaction amplification

The PCR amplification was performed in a volume of 25 μL containing 1 μL of genomic DNA at a concentration of 0.1 μM, 1 μL of forward primer at a concentration of 0.1 μM, 1 μL of reverse primer at a concentration of 0.1 μM, 2.5 μL of dNTP Mix (2 mM each) (Thermo Fisher Scientific, USA), 2.5 μL 10× Tag Buffer (Thermo Fisher Scientific), 1.5–2.5 μL 25 mM magnesium chloride (MgCl) (Thermo Fisher Scientific), 0.5 μL Tag DNA polymerase (Thermo Fisher Scientific), and mQ up to 25 μL. Amplification was carried out in a T100 thermal cycler (Bio-Rad, USA) under the following conditions: Initial denaturation at 95°C for 5 min, then 30 denaturation cycles at 95°C for 1 min, annealing at 62–70°C for 1 min for Roe deer and 52–60°C for Siberian stag and Saiga, elongation at 72°C for 1 min, and final elongation at 72°C for 10 min. The PCR amplification products were purified using the QIAquick PCR Purification Kit (Qiagen), according to the manufacturer’s instructions.

### Sequencing of PCR products

Sequencing of PCR products was performed using BigDye Terminator v3.1 kit (Applied Biosystems, USA), with primers used for PCR amplification according to the manufacturer’s instructions. The separation of sequencing products was carried out on a DNA analyzer 3730 × l genetic analyzer. The contig was assembled in LaserGene software (https://www.dnastar.com/software/lasergene/). Identification was performed using the NCBI web resource (https://blast.ncbi.nlm.nih.gov/Blast.cgi).

## Results

As a result of primer design, three specific pairs were selected, as shown in Table-2. DNA sizes amplified by primers for Roe deer, Saiga, and the Siberian stag were 542, 587, and 525 bp, respectively.

**Figure-1:**
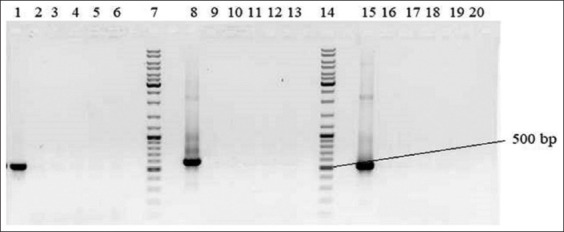
DNA amplification of Roe deer, Saiga, Siberian stag, argali, sheep, and cow by polymerase chain reaction based on the developed primers. Lanes 1, 9, and 16: Roe deer; lanes 2, 8, and 17: Saiga; lanes 3, 10, and 15: Deer; lanes 4, 11, and 18: Argali; lanes 5, 12, and 19: Sheep; lanes 6, 13, and 20: Cow; and lanes 7 and 14: Molecular marker.

The polymerase reaction was optimized to increase the specificity of amplification. The most effective parameters for amplifying the Cyt b fragment of Roe deer were a 1.5 mM MgCl concentration in the reaction mixture and an annealing temperature of 61°C. The optimal parameters for Siberian stag and Saiga were 2.5 mM concentration of MgCl in the reaction mixture and an annealing temperature of 55°C. The PCR with DNA from other animal species did not show product formation (Figure-1). The length of the amplified products corresponds to the predicted size. Sequencing the amplified products and subsequent comparison with the corresponding reference sequence showed similarities ranging from 99.99% to 100%. When determining the specificity of PCR, primers for one animal species were designed based on the material from other species of artiodactyl animals.

**Figure-2:**
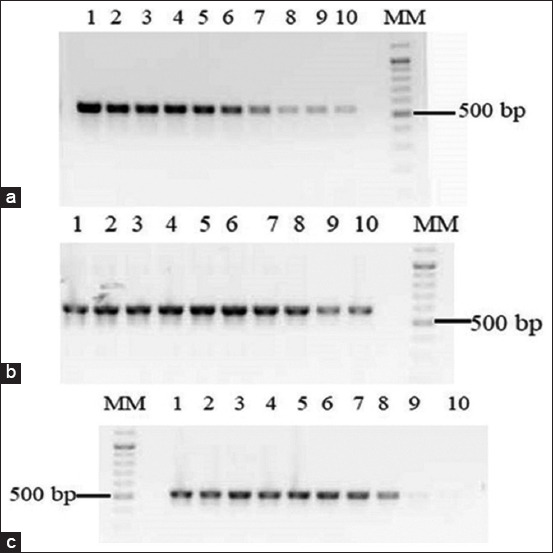
DNA amplification of (a) Roe deer, (b) Saiga, and (c) Siberian stag by polymerase chain reaction. Lane 1: 1000 pg; lane 2: 500 pg; lane 3: 250 pg; lane 4: 125 pg; lane 5: 62.5 pg; lane 6: 31.2 pg; lane 7: 15.6 pg; lane 8: 7.5 pg; lane 9: 3.9 pg; lane 10: 1.9 pg; and lane MM=Molecular marker.

The sensitivity of the quantitative PCR reaction was tested using serial dilutions of DNA isolated from biological materials of artiodactyl animals. The PCR detection limit ranged from 15.6 pg to 1.9 pg of DNA in 25 mL of the reaction mixture (Figure-2).

## Discussion

The protection of wild artiodactyl animals, which are an important part of biodiversity, is a priority task for the conservation of genetic resources of rare and endangered species of the fauna in many countries of the world. To regulate human activities in the field of trade in products of animal origin, the veterinary, sanitary, and food legislation of the European Union includes several thousand regulations governing the veterinary and sanitary requirements of trade in animals and food of animal origin [19]. Despite the existence of legislative acts and various agreements on the trade of animals, poaching continues to cause significant damage to the population of wild artiodactyls. The main reason for the low efficiency of law enforcement is the lack of reliable methods for identifying animal remains by morphological features. Unlike morphological and spectrometric methods, modern DNA test systems make it possible to identify an animal species reliably [20]. Methods based on molecular markers are most widely used for identifying food and processed animal products. The advantage of molecular markers is associated with the need for small amounts of DNA, the ability to identify an animal by several markers, and the use of a PCR method that allows obtaining both qualitative and quantitative results. The most popular markers for tracking and controlling products of animal origin, such as SSR and SNP, are highly informative and can identify animals between species and within species [21].

To identify animal species, a standardized Cyt b gene has been proposed. This marker proved to be effective in identifying animals in epidemic conditions and illegal trade in wild animals. The mammalian Cyt b gene is the least susceptible to mutations that change the amino acid sequence of a protein, rendering it suitable for identifying unique genetic sequences. Multiplex PCR analysis based on four pairs of primers for the gene of buffalo, cattle, pig, and duck made it possible to determine falsification in all types of food products with high accuracy [22]. Using the Cyt b gene and direct PCR, a rare paka animal was identified in the illegal meat trade. The method based on a single-nucleotide substitution of the Cyt b gene proved effective and differentiated paka meat with 100% certainty [14]. The presented results demonstrate the usefulness of PCR analysis based on primers for the Cyt b gene in the authentication of meat and meat products in the laboratory. Applying modern developments in the field of high-speed polymerases render identifying an animal species in the field a real possibility [23].

To identify Roe deer, Saiga, and Siberian stag living in Kazakhstan based on biological samples, three pairs of primers were designed to amplify fragments of the Cyt b genes of these animal species. The developed primers identified a fragment of the Cyt b gene with a size of 525–587 bp, which significantly differs in nucleotide composition in the studied animal species. The main reason for using mitochondrial DNA for species identification is associated with several gene properties: The absence of gene recombination, significant compartmentalization, the absence of introns, and resistance to biodegradation [24]. Due to the conservatism of mitochondrial DNA, a number of its sequences are used as genetic markers. According to Kumar *et al*. [25], analysis of the mitochondrial DNA Cyt b gene by PCR-RFLP differentiated the meat of closely related animal species in food. At the same time, the method demonstrated high accuracy and specificity in research.

PCR analysis was performed using the obtained pairs of primers to identify Roe deer, Saiga, and Siberian stag from biological material. The species origin of each sample was confirmed by comparing the test sequence with reference sequences. The similarity of the studied samples with the corresponding reference sequence ranged from 99.99% to 100%, and the PCR sensitivity ranged from 15.6 pg to 1.9 pg DNA, indicating the suitability of the developed primers for species identification of wild artiodactyl animals.

## Conclusion

Preservation of the number of wild artiodactyl animals is an urgent task for Kazakhstan. To identify Roe deer, Saiga, and Siberian stag from biological samples, three pairs of primers were developed to amplify fragments of the Cyt b gene ranging in size from 525 to 587 bp. Analysis of amplified and reference sequences showed high genetic similarity of the amplified and reference sequences indicate the specificity of the designed primers. This PCR method, which was optimized to determine the Cyt b gene of Roe deer, Saiga, and Siberian stag in biological samples in the laboratory, can also be used to detect Roe deer, Saiga, and Siberian stag meat in food adulteration.

## Authors’ Contributions

KM and YR: Contributed to conceptualization and design of the study. DK, SZ, and KT: Sampling, DNA extraction, PCR optimization, and sequencing. AS, and KMukanov: Troubleshooting of PCR technique and helped in analysis of PCR results. KM and AS: Analyzed and interpreted the data. KM: Prepared and wrote the original draft. KMukanov and YR: Reviewed and edited the manuscript. All authors have read and approved the final manuscript.

## References

[1] Goudaa S, Kerry R.G, Dasc A, Chauhana N.S (2020). Wildlife forensics:A boon for species identification and conservation implications. Forensic Sci. Int.

[2] Ferreira P.B, Torres R, Garcia J.E (2011). Single nucleotide polymorphisms from cytochrome b gene as a useful protocol in forensic genetics against the illegal hunting of manatees:*Trichechus manatus*, *Trichechus inunguis*, *Trichechus senegalensis*, and *Dugong dugon* (*Eutheria*:*Sirenia*). Zoologia (Curitiba Impresson).

[3] Rodionov A, Deniskova T, Dotsev A, Volkova V, Petrov S, Kharzinova V, Koshkina O, Abdelmanova A, Solovieva A, Shakhin A, Bardukov N, Zinovieva N (2021). Combination of multiple microsatellite analysis and genome-wide SNP genotyping helps to solve wildlife crime:A case study of poaching of a caucasian tur (*Capra caucasica*) in Russian mountain national park. Animals (Basel).

[4] Ghosh T, Sharma A, Mondol S (2021). Optimisation and application of a forensic microsatellite panel to combat Greater-one horned rhinoceros (*Rhinoceros unicornis*) poaching in India. Forensic Sci. Int. Genet.

[5] Zdunczyk Z, Minakowski D, Frejnagel S, Flis M (1999). Comparative study of the chemical composition and nutritional value of pumpkin seed cake, soybean meal and casein. Nahrung.

[6] Parson W, Pegoraro K, Niederstätter H, Föger M, Steinlechner M (2000). Species identification by means of the cytochrome b gene. Int. J. Legal Med.

[7] Lopez-Oceja A, Gamarra D, Borragan S, Jiménez-Moreno S, De Pancorbo M.M (2016). New cyt b gene universal primer set for forensic analysis. Forensic Sci. Int. Genet.

[8] Ewart K.M, Frankham G.J, McEwing R, Webster L.M.I, Ciavaglia S.A, Linacre A.M.T, The D.T, Ovouthan K, Johnson R.N (2018). An internationally standardized species identification test for use on suspected seized rhinoceros horn in the illegal wildlife trade. Forensic Sci. Int. Genet.

[9] Ghosh A, Basu S, Jabin G, Khatri H, Singh S.K, Maheswaran G, Chandra K, Thakur M (2019). Wildlife forensics in voiding false offences:A case study to deal with unidentified cooked meat. Forensic Sci. Int.

[10] Pfeiffer I, Burger J, Brening B (2004). Diagnostic polymorphisms in the mitochondrial cytochrome b gene allow discrimination between cattle, sheep, goat, roe buck and deer by PCR-RFLP. BMC Genet.

[11] Nesvadbová M, Bořilová G, Hulánková R (2019). PCR-RFLP identification of meat from red deer, sika deer, roe deer, fallow deer, mouflon, wild boar, hare and cattle. Acta Vet. Brno.

[12] Naidu A, Fitak R.R, Munguia-Vega A, Culver M (2012). Novel primers for complete mitochondrial cytochrome b gene sequencing in mammals. Mol. Ecol. Resour.

[13] Muangkram Y, Wajjwalku W, Amano A, Sukmak M (2018). The novel primers for mammal species identification-based mitochondrial cytochrome b sequence:Implication for reserved wild animals in Thailand and endangered mammal species in Southeast Asia. Mitochondrial DNA A DNA Mapp. Seq. Anal.

[14] Silva-Neto A.A, Ferreira P.B, Torres R.A, Texeira R.H.F, Duarte J.M.B, Barbosa A.C, Vargas R.C, Garcia J.E (2016). Diagnostic cytochrome b gene profiles for the identification of paca (*Cuniculus paca*) bushmeat:Implications for the monitoring of illegal hunting and wildlife trade. Braz. J. Biol.

[15] Bataille M, Crainic K, Leterreux M, Durigon M, De Mazancourt P (1999). Multiplex amplification of mitochondrial DNA for human and species identification in forensic evaluation. Forensic Sci. Int.

[16] Andrejevic M, Markovic M.K, Bursac B, Mihajlovic M, Tanasic V, Kecmanovic M, Keckarevic D (2019). Identification of a broad spectrum of mammalian and avian species using the short fragment of the mitochondrially encoded cytochrome b gene. Forensic Sci. Med. Pathol.

[17] Hsieh H.M, Chiang H.L, Tsai L.C, Lai S.Y, Huang N.E, Linacre A, Lee J.C (2001). Cytochrome b gene for species identification of the conservation animals. Forensic Sci. Int.

[18] Hall T (1999). Bioedit:A user-friendly biological sequence alignment editor and analysis program for windows 95/98/NT. Nucl. Acids. Symp. Ser.

[19] Bondoc I (2016). European Regulation in the Veterinary Sanitary and Food Safety Area, a Component of the European Policies on the Safety of Food Products and the Protection of Consumer Interests:A 2007 Retrospective. Part Two:Regulations. European Law in the Veterinary Sanitary and Food Safety Area:Exhaustive Study for the Period 2007–2017.

[20] Eaton M.J, Meyers G.L, Kolokotronis S.O, Leslie M.S, Martin A.P, Amato G (2010). Barcoding bushmeat:Molecular identification of central African and South American harvested vertebrates. Conserv. Genet.

[21] Fanelli V, Mascio I, Miazzi M.M, Savoia M.A, De Giovanni C, Montemurro C (2021). Molecular approaches to agri-food traceability and authentication:An updated review. Foods.

[22] Wang L, Hang X, Geng R (2019). Molecular detection of adulteration in commercial buffalo meat products by multiplex PCR assay. Food Sci. Technol.

[23] Song K.Y, Hwang H.J, Kim J.H (2017). Ultra-fast DNA-based multiplex convection PCR method for meat species identification with possible on-site applications. Food Chem.

[24] Linacre A, Tobe S.S (2011). An overview to the investigative approach to species testing in wildlife forensic science. Investig Genet.

[25] Kumar D, Singh S.P, Karabasanavar N.S, Singh R, Umapathi V (2014). Authentication of beef, carabeef, chevon, mutton, and pork by a PCR-RFLP assay of mitochondrial cytb gene. J. Food Sci. Technol.

